# Norrin Protects Retinal Ganglion Cells from Excitotoxic Damage via the Induction of Leukemia Inhibitory Factor

**DOI:** 10.3390/cells9020277

**Published:** 2020-01-23

**Authors:** Stefan Kassumeh, Stephanie Leopold, Rudolf Fuchshofer, Carina N. Thomas, Siegfried G. Priglinger, Ernst R. Tamm, Andreas Ohlmann

**Affiliations:** 1 Laboratory for Cell and Molecular Biology, Department of Ophthalmology, University Hospital, LMU Munich, 80336 Munich, Germany; Stefan.Kassumeh@med.uni-muenchen.de (S.K.); Siegfried.Priglinger@med.uni-muenchen.de (S.G.P.); 2 Institute of Human Anatomy and Embryology, University of Regensburg, 93053 Regensburg, Germany; stephi.leopold@gmx.de (S.L.); Rudolf.Fuchshofer@vkl.uni-regensburg.de (R.F.); Ernst.Tamm@vkl.uni-regensburg.de (E.R.T.); 3 Wellman Center for Photomedicine, Massachusetts General Hospital, Harvard Medical School, Boston, MA 02114, USA; CNTHOMAS@mgh.harvard.edu

**Keywords:** Lif, retinal ganglion cells, neuroprotection, excitotoxic damage, NMDA, apoptosis, Norrin, Wnt/β-catenin signaling

## Abstract

Purpose: To investigate whether and how leukemia inhibitory factor (Lif) is involved in mediating the neuroprotective effects of Norrin on retinal ganglion cells (RGC) following excitotoxic damage. Norrin is a secreted protein that protects RGC from *N-*methyl-d-aspartate (NMDA)-mediated excitotoxic damage, which is accompanied by increased expression of protective factors such as Lif, Edn2 and Fgf2. Methods: Lif-deficient mice were injected with NMDA in one eye and NMDA plus Norrin into the other eye. RGC damage was investigated and quantified by TUNEL labeling 24 h after injection. Retinal mRNA expression was analyzed by quantitative real-time polymerase chain reaction following retinal treatment. Results: After intravitreal injection of NMDA and Norrin in wild-type mice approximately 50% less TUNEL positive cells were observed in the RGC layer when compared to NMDA-treated littermates, an effect which was lost in Lif-deficient mice. The mRNA expression for Gfap, a marker for Müller cell gliosis, as well as Edn2 and Fgf2 was induced in wild-type mice following NMDA/Norrin treatment but substantially blocked in Lif-deficient mice. Conclusions: Norrin mediates its protective properties on RGC via Lif, which is required to enhance Müller cell gliosis and to induce protective factors such as Edn2 or Fgf2.

## 1. Introduction

In the retina, light is perceived by photoreceptors, which transmit their visual information via horizontal, amacrine and bipolar cells for initial neuronal processing to retinal ganglion cells (RGC), which are located in the inner retina. After further information processing, RGC project their axons to the brain after converging at the optic disc to form the optic nerve [1]. The importance of RGC for visual function is pointed out in diseases such as glaucoma or Leber’s hereditary optic neuropathy leading to degeneration of RGC and subsequent blindness [2]. In glaucoma, intraocular pressure (IOP) is the leading risk factor for disease progression and unfortunately IOP lowering is the only therapeutic option so far. Despite sufficient IOP regulation, some patients experience a further disease progression, suggesting that additional mechanisms might contribute to glaucomatous RGC degeneration [3]. Thus, neuroprotective strategies to protect RGC against glaucomatous damage have been discussed for the past decades. To investigate protective mechanisms or potential therapeutic options, inter alia, the animal model of N-methyl-D-aspartate (NMDA) induced apoptosis of RGC has been established [4]. In the retina, NMDA induces excitotoxicity of RGC and subtypes of amacrine cells by hyperactivation of NMDA-type glutamate receptors, which results in an excessive Ca^2+^ influx that subsequently propagates proapoptotic signaling cascades [5,6].

The Wnt/β-catenin signaling pathway is a key mechanism for stem cell maintenance, cell proliferation, cell differentiation and adult tissue homeostasis. Thus, mutations in this pathway can affect embryonal development and cancer biology, which may lead to several other diseases. Wnt proteins are secreted glycolipoproteins binding to frizzled receptors (FZD). The main downstream signaling molecule in Wnt signaling is β-catenin, which is constitutively expressed in cells. Without activation by Wnt proteins, β-catenin will be continuously degraded by its degradation complex. For activation, Wnt ligands bind to FZDs and form a complex with the co-receptor low-density lipoprotein receptor related protein (LRP)5 or 6, resulting in an inactivation of the β-catenin degradation complex. Subsequently, β-catenin accumulates in the cytoplasm, translocates into the nucleus and then induces the expression of the differentially expressed target gene [7].

In the retina, Wnt/β-catenin signaling inter alia induces retinal pigment epithelium (RPE) morphogenesis and pigmentation [8], the development of the retinal vasculature, as well as the formation of the inner blood-retinal barrier [9] and mediates protective effects on damaged retinal neurons. One predominant signaling molecule that can activate canonical Wnt signaling via binding to FZD4 is Norrin. In Norrin- or FZD4- deficient mice, a retarded development of the superficial vascular plexus, a complete lack of intraretinal vasculature and an impaired blood-retina barrier was observed [10,11]. Vice versa, transgenic overexpression of Norrin induces vascular regrowth into vaso-obliterated areas. This could be shown for an oxygen-induced retinopathy, which is the model for retinopathy of prematurity in mice [12]. In addition to its effect on retinal vasculature, transgenic overexpression in mice also significantly increases the numbers of retinal progenitor cells [7]. However, in Norrin-deficient mice, a continuous loss of cells in the retinal ganglion cell layer was observed [13,14]. Intriguingly, recent studies indicate that the expression of Norrin increases dendritic arborization as well as dendritic length and spine density of cortical neurons [15]. Furthermore, following induction of an oxygen-induced retinopathy, Norrin protects RGC against degeneration [16]. In previous studies, we observed a protective effect of Norrin on RGC and photoreceptors after acute or chronic damage [17,18]. For instance, following induction of excitotoxic RGC damage by intravitreal injection of N-methyl-D-aspartate (NMDA), the additional application of Norrin enhances reactive gliosis of Müller cells, which in turn leads to an enhanced expression of protective factors such as leukemia inhibitory factor (Lif), fibroblast growth factor (Fgf)2 and endothelin (Edn)2 [12]. Furthermore, for Müller cell gliosis following light-induced damage of photoreceptors, a self-enforcing feedback loop is described. Concisely, after damage on photoreceptors, Müller cells induce the expression of Lif, which in turn enhances the expression of Edn2 in photoreceptors. Subsequently, Edn2 further accelerates the expression of Lif in Müller cells, which leads to an increased reactive Müller cell gliosis and an induction of protective factors such as Fgf2 [19].

Lif is a secreted protein, which belongs to the interleukin-6 family of cytokines, as well as the ciliary neurotrophic factor (Cntf) [20]. After binding to the heterodimeric Lif receptor-β/gp130 receptor complex, Lif can activate Ras/MAPK/ERK, PI3K/Akt and JAK/STAT signaling to mediate stimulating or inhibiting effects on cell proliferation, differentiation and survival. Besides other functions, in the central nervous system, Lif is involved in differentiation of neurons and glia cells, increases neuronal stem cell population and modulates neuroinflammatory processes after injury [21]. In the retina, Lif and its receptors play a key role in retinal differentiation during development [22] and for protection of retinal neurons in degenerating retina [23]. Further on, in a recent study, Yang and colleagues demonstrated that Lif protects vascular integrity and prevents RGC degeneration in retinae of diabetic mice [24].

Since Norrin induces the expression of Lif, we investigated whether Norrin mediates its effects on Müller cells and retinal neurons via an induction of Lif as a central downstream signaling molecule. To prove our hypothesis, NMDA was injected into the vitreous cavity of homozygous (Lif^−/−^) and heterozygous (Lif^+/−^) Lif-deficient mice as well as wild-type littermates to induce excitotoxic damage in RGC, either with or without Norrin. Apoptotic cells in the RGC layer and inner nuclear layer (INL) were detected by TUNEL assay and quantified. Furthermore, reactive Müller cell gliosis and the expression of protective factors (Gfap, Edn2, Fgf2) were investigated following intravitreal treatment with NMDA and Norrin.

## 2. Materials and Methods

### 2.1. Animals

In this study, the animal procedures complied with the regulations of the Association for Research in Vision and Ophthalmology (ARVO) for the use of animals in ophthalmic research and were authorized by the local authorities (Regierung der Oberpfalz, Bavaria, Germany; project identification code—54-2532.1-37/12; date of approval—05.12.2012).

Lif-deficient mice in the C57BL/6 background were provided by Dr. M. Sendtner (University of Würzburg, Würzburg, Germany) [25]. Animals were housed in the animal facility of the University of Regensburg with a controlled light cycle of 12 h and were given food as well as water ad libitum. In the following experiments, 6 to 12 weeks old homozygous (*Lif^−/−^*) and heterozygous Lif-deficient mice (*Lif^+/−^*) and wild-type littermates were used.

For genotyping of the Lif-deficient mice, the neomycin cassette was amplified by polymerase chain reaction (PCR) with the primer pairs 5′-CCAGCTCTTCAGCAATATCACGGG-3′ and 5′-CCTGTCCGGTGCCCTGAATGAACT-3′, leading to an DNA amplicon of a 514 bp. For identification of wild-type mice, PCR analysis was performed with the primer pair 5′-CGCCTAACATGACAGACTTCCCAT-3′ and 5′-AGGCCCCTCATGACGTCTATAGTA-3′, amplifying a 192 bp DNA fragment of the Lif wild-type gene locus. DNA amplification was performed as follows—initial denaturation at 94 °C for 10 min, followed by 35 cycles with the program 94 °C for 30 s for denaturation, annealing at 68 °C for 45 s and extension at 72 °C for 45 s. All primers were obtained from Thermo Fisher Scientific (Thermo Fisher Scientific Inc., Waltham, MA, USA).

For preparation of the eyes, mice were anesthetized with isoflurane and sacrificed by cervical dislocation before enucleation.

### 2.2. NMDA-Induced Damage on Retinal Ganglion Cells

Excitotoxic damage of retinal ganglion cells was induced by NMDA as previously described [17]. Concisely, 8-week-old mice were anesthetized with isoflurane. Subsequently, 3 µL phosphate-buffered saline (PBS) were injected into the vitreous cavity of one eye and 3 µL NMDA [10 mM, Merck Millipore, Burlington, MA, USA] into the fellow eye or 3 µL NMDA in one and 3 µL NMDA plus Norrin [5 ng/mL] into the other eye. To allow for intraocular pressure equalization, the injection needle (32 Gauge) was kept in the vitreous cavity for an additional 20 s after injection.

### 2.3. Overexpression and Purification of Human Recombinant Norrin

The synthesis and purification of human recombinant Norrin was performed as described previously [12]. For this, transfected EBNA-293-HEK cells were cultivated in Dulbecco’s Modified Eagle’s Medium (DMEM) supplemented with 5% fetal calf serum (FCS), 20 µg/mL gentamicin, 250 µg/mL geneticin (G418) and 300 µg/mL hygromycin (all obtained from Thermo Fisher Scientific). To harvest recombinant Norrin, cells were starved in serum-depleted cell culture medium for 3 days. Purification of human recombinant Norrin was performed by affinity chromatography using heparin agarose (Merck Millipore). Specificity and purity of the isolated protein was analyzed by SDS-polyacrylamide gel electrophoresis followed by a subsequent silver staining or western blot analysis according to standard protocols.

### 2.4. RNA Isolation, cDNA Synthesis and Real-Time rt-PCR Analysis

For mRNA expression analysis, the total RNA of retinae from treated mice was isolated with peqGOLD TriFast (Peqlab Biotechnology, Erlangen, Germany) according to the manufacturers’ instructions. The RNA concentration and the OD260/OD280 ratio were measured with the NanoDrop-1000 spectrophotometer (Peqlab Biotechnology). Only an OD ratio between 1.6 and 2.0 was considered suitable for single-strand DNA synthesis, which was performed by using the iScript cDNA Synthesis Kit (Bio-Rad Laboratories Inc., Hercules, CA, USA) in accordance with the manufacturers’ recommendations. Real-time-PCR was conducted on a CFX real-time PCR detection system (Bio-Rad Laboratories Inc.). Polymerase chain reaction was performed in a volume of 15 µL using the 2x SYBR Green Master Mix (Bio-Rad). The thermocycler profile was 40 cycles at 95 °C for 10 s for denaturation and 40 s of annealing and extension at 60 °C. All primer pairs of the candidate genes span exon-intron boundaries (Table 1) and were obtained from Thermo Fisher Scientific. *Gnb2l* served as housekeeper for relative quantification. Results were analyzed using the iQ Optical System Software Version 2.1 (Bio-Rad).

### 2.5. Light Microscopy

Following preparation, eyes were fixed in 2.5% glutaraldehyde (SERVA) and washed in cacodylate buffer. After post-fixation in osmium tetroxide (Carl Roth AG, Arlesheim, Switzerland), specimens were embedded in Epon (Carl Roth AG) in accordance to standard protocols. Semithin meridional sections (1 µm) of eyes and transverse sections of optic nerves were performed. All specimens were stained with paraphenylenediamine or Richardson’s stain, respectively, according to standard protocols [26,27]. Image acquisition was performed on Axio Imager using the Axiovision software 4.8 (Carl Zeiss AG, Oberkochen, Germany).

### 2.6. TUNEL Labeling

For TdT-mediated dUTP-biotin nick end labeling (TUNEL), eyes were fixed in 4% paraformaldehyde (Carl Roth) for 4 h and embedded in paraffin, which is in accordance with standard procedures. After preparation of meridional sections, the TUNEL assay was performed in accordance with the manufacturer’s instructions (Deadend Fluorometric TUNEL system, Promega, Madison, WI, USA), mounted in fluorescent mounting medium containing 1:10 DAPI (Sigma-Aldrich, St. Louis, MO, USA) and analyzed on an Axio Imager fluorescence microscope with an integrated apotome module using the Axiovision software 4.8 (Carl Zeiss AG). For quantification, the number of apoptotic cells in the retinal ganglion cell layer and inner nuclear layer was counted and calculated as the number of TUNEL positive cells per 1000 µm using the Axiovision software 4.8.

### 2.7. Statistical Analysis

All results are expressed as mean ± SEM. A one-way ANOVA was performed to compare the mean variables of more than 2 groups. An LSD or Games Howell post-hoc test followed for data that meet or do not meet the criteria of the assumption of homogeneity of variances, respectively. P-values less than 0.05 were considered to be statistically significant.

## 3. Results

### 3.1. Lif-Deficient Mice do not Show an Obvious Retinal or Optic Nerve Phenotype

To investigate possible morphological changes of the retina and optic nerve in Lif-deficient mice, meridional semithin sections of wild-type, heterozygous and homozygous mice were investigated.

**Figure 1:**
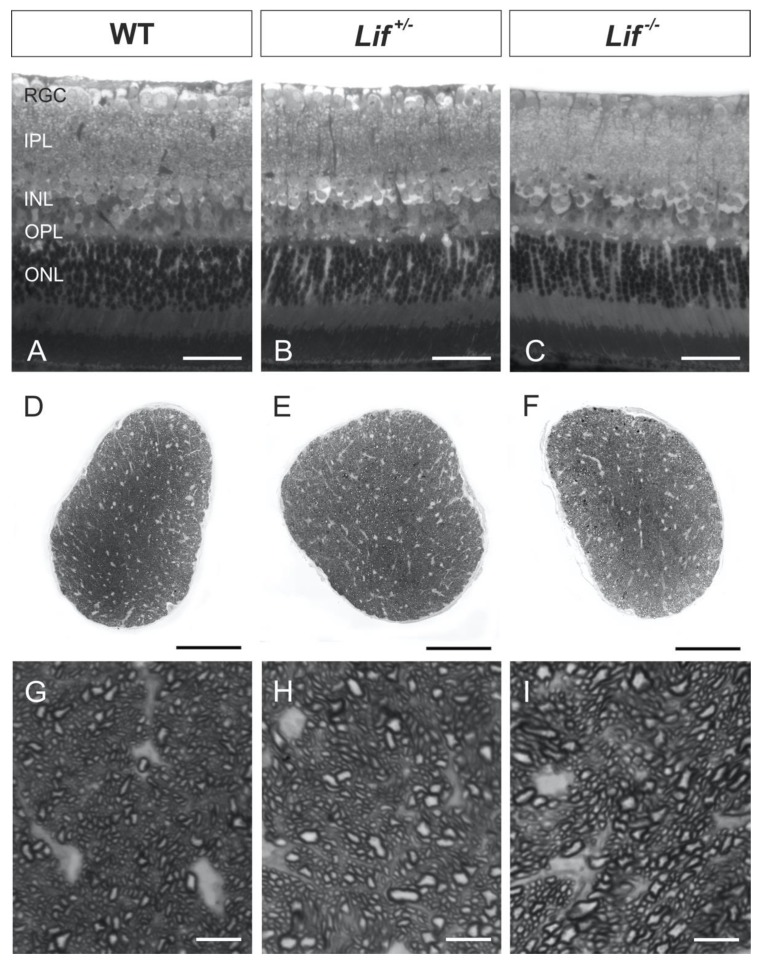
Lif-deficient mice do not show an obvious retinal or optic nerve phenotype. Representative semithin sections of retinae (**A**–**C**) and optic nerves (**D**–**I**) from adult hetero- (**B**,**E**,**H**) and homozygous (**C**,**F**,**I**), Lif-deficient mice and wild-type littermates (**A**,**D**,**G**). Scale bars: **A**–**C**, 50 µm; **D**–**F**, 100 µm; **G**–**I**, 10 µm; RGC: retinal ganglion cell layer; IPL: inner plexiform layer; INL: inner nuclear layer; OPL: outer plexiform layer; ONL: outer nuclear layer.

In meridional retinal sections of all genotypes, no morphological alterations in the retinal architecture were observed (Figure 1A–C). In particular, no changes in the thickness and number of cells in the RGC layer between hetero- and homozygous Lif-deficient mice and wild-type controls were detected. Furthermore, the cross-sections of the optic nerves of all three genotypes revealed a normal distribution of glial tissue and axonal bundles (Figure 1D–F). Only a few dark stained spots were observed, indicating myelin whorls from degenerating axons. In addition, no differences in the number and size of RGC axons between hetero- (*Lif^+/−^*) and homozygous Lif-deficient mice (*Lif^−/−^*) as well as wild-type littermates were observed (Figure 1G–I). Overall, by histological analysis of retinae and optic nerves from homozygous and heterozygous, Lif-deficient mice, no distinct morphological phenotype was observed.

### 3.2. Norrin Induces Lif Expression in Retinae Following Excitotoxic Damage of Retinal Neurons

To investigate Norrin-mediated enhancement of Lif expression after excitotoxic damage of retinal neurons, retinal mRNA levels of Lif were analyzed by real-time RT-PCR.

**Figure 2:**
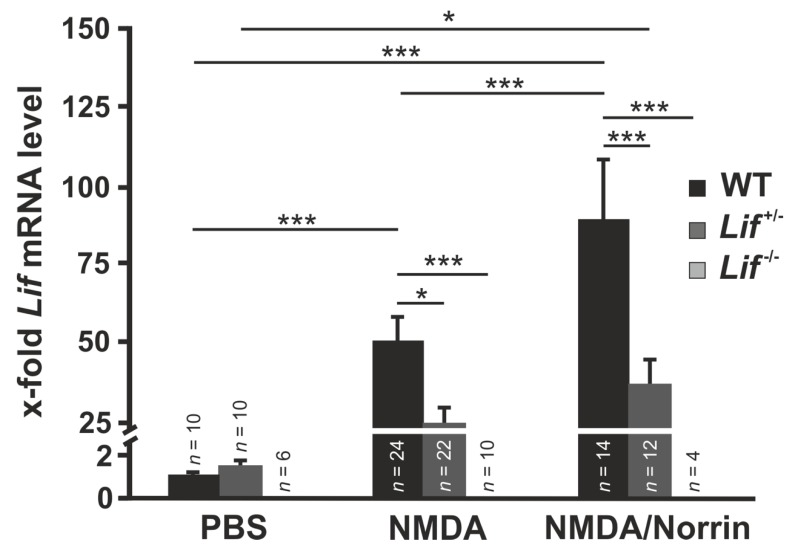
Norrin enhances N-methyl-D-aspartate (NMDA) induced Lif expression in mice retinae. **Real-time** RT-PCR on retinal total RNA for *Lif* from heterozygous (*Lif^+/−^*) and homozygous, Lif-deficient mice (*Lif^−/−^*), as well as wild-type (WT) littermates 7 h after intravitreal injection of 3 µL PBS, 3 µL NMDA [10 mM] or 3 µL NMDA [10 mM] with Norrin [5 ng/µL]. * *p* < 0.05; *** *p* < 0.001.

In response to intravitreal injection of NMDA in wild-type mice, retinal mRNA levels of Lif were substantially increased (49.3 ± 9.2) when compared to PBS treated controls. This effect was further enhanced when NMDA was injected in combination with Norrin (89.6 ± 18.3; Figure 2). However, in *Lif^+/−^* mice, the mRNA levels of Lif decreased after NMDA (24.5 ± 5.5) and NMDA with Norrin injection (37.0 ± 8.2) when compared to wild-type controls (Figure 2). No Lif mRNA was detected in homozygous Lif-deficient mice (Figure 2).

### 3.3. Norrin Mediates Its Neuroprotective Effect via an Induction of Lif

To analyze if Lif is a central downstream mediator of Norrin-mediated neuroprotective effects on retinal neurons, TUNEL assays on retinal meridional sections from hetero- and homozygous Lif-deficient mice were performed 24 h after intravitreal injection of Norrin and/or NMDA.

**Figure 3:**
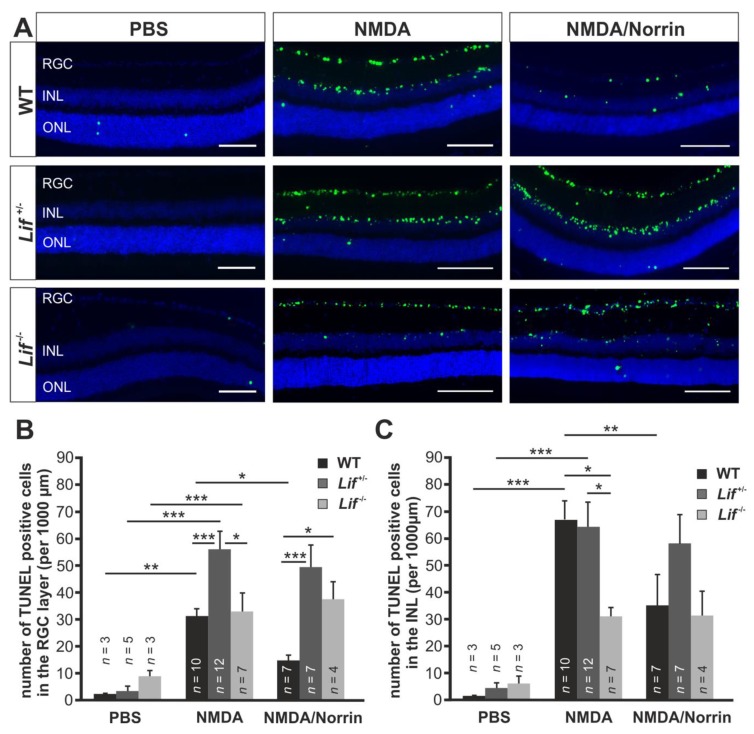
Norrin mediates its neuroprotective effect via an induction of Lif. (**A**) Representative TUNEL staining (green) of retinae from heterozygous (*Lif^+/−^*) and homozygous, Lif-deficient mice (*Lif^−/−^*), as well as wild-type (WT) littermates 24 h after intravitreal injection of 3 µL PBS, 3 µL NMDA [10mM] or 3 µL NMDA [10mM] with Norrin [5 ng/µL]. RGC: retinal ganglion cell layer; INL: inner nuclear layer; ONL: outer nuclear layer; blue, DAPI staining; scale bars: 50 µm. (**B**,**C**). The number of TUNEL positive cells in the RGC (**B**) and INL (**C**) of all genotypes was quantified and plotted as the number of TUNEL positive cells per 1000 µm retinal length. * *p* < 0.05; ** *p* < 0.01; *** *p* < 0.001.

In wild-type mice many TUNEL-positive cells in the retinal ganglion cell layer were observed following intravitreal injection of NMDA. When NMDA was injected in combination with Norrin, this effect was substantially reduced (Figure 3A). Quantification of apoptotic neurons in the RGC layer showed more than 30 TUNEL-positive cells per 1,000 µm retinal length (31.8 ± 3.1) in NMDA-treated retinae. The number of TUNEL-positive cells was substantially reduced to 14.8 ± 3.5 when NMDA and Norrin were injected (Figure 3B). Further on, in heterozygous mice, the number of TUNEL-positive cells in the RGC layer was significantly increased by 58.1 ± 6.6 per 1000 µm retinal length and approximately twice as much as in NMDA-treated wild-type littermates (Figure 3A,B). However, following treatment of *Lif^+/−^* mice with NMDA and Norrin, only a small reduction of apoptotic cells to 51.5 ± 8.0 per 1000 µm retinal length was detected in the RGC layer (Figure 3A,B). Interestingly, treatment of homozygous Lif-deficient mice with NMDA led to a substantial number of TUNEL-positive cells in the RGC layer (33.9 ± 7.1 per 1000 µm length; Figure 3A,B), which was similar to that of wild-type controls and approximately less than 60% of that observed in *Lif^+/−^* mice. However, the additional injection of Norrin had no effect on the number of TUNEL-positive neurons in the RGC layer of *Lif^−/−^* mice (38.8 ± 6.6 per 1000 µm length; Figure 3A,B).

Since specific subtypes of amacrine cells express the NMDA receptor and hence are affected by NMDA treatment, the number of apoptotic cells in the inner plexiform layer (INL) of several genotypes was analyzed. In wild-type mice, numerous TUNEL-positive cells (67.8 ± 6.6 per 1000 µm retinal length) in the INL were observed after treatment with NMDA, which was significantly lower when the eyes were injected with the combined treatment (34.5 ± 12.3; Figure 3A,C). However, in homozygous, Lif-deficient mice, 31.0 ± 3.5 TUNEL-positive cells per 1000 µm retinal length were detected, which was equivalent to that of wild-type mice. Moreover, in heterozygous, Lif-deficient mice, the number of TUNEL positive cells (64.4 ± 9.1 per 1000 µm) was approximately twice as high as in wild-type controls and homozygous Lif-deficient mice (Figure 3A,C). As described for the RGC layer, the combined injection of NMDA with Norrin had no effect on the number of apoptotic cells in the INL of hetero- (58.4 ± 10.6 per 1000 µm) or homozygous (31.5 ± 9.1 per 1000 µm) Lif-deficient mice (Figure 3A,C).

### 3.4. Norrin Mediates Müller Cell Gliosis via LIF Signaling

Retinal damage usually induces gliosis reaction of Müller cells, which can lead to an expression of protective factors as well as proapoptotic signaling molecules [28]. In a previous study, we could demonstrate that Norrin enhances gliosis reaction of Müller cells, leading to an increased expression of neuroprotective factors [17]. To find out if the expression of Lif is required to mediate the Norrin-induced gliosis reaction of Müller cells, the mRNA level for Gfap, a marker for Müller cell gliosis, was analyzed in hetero- and homozygous Lif-deficient mice following treatment with Norrin and/or NMDA.

**Figure 4:**
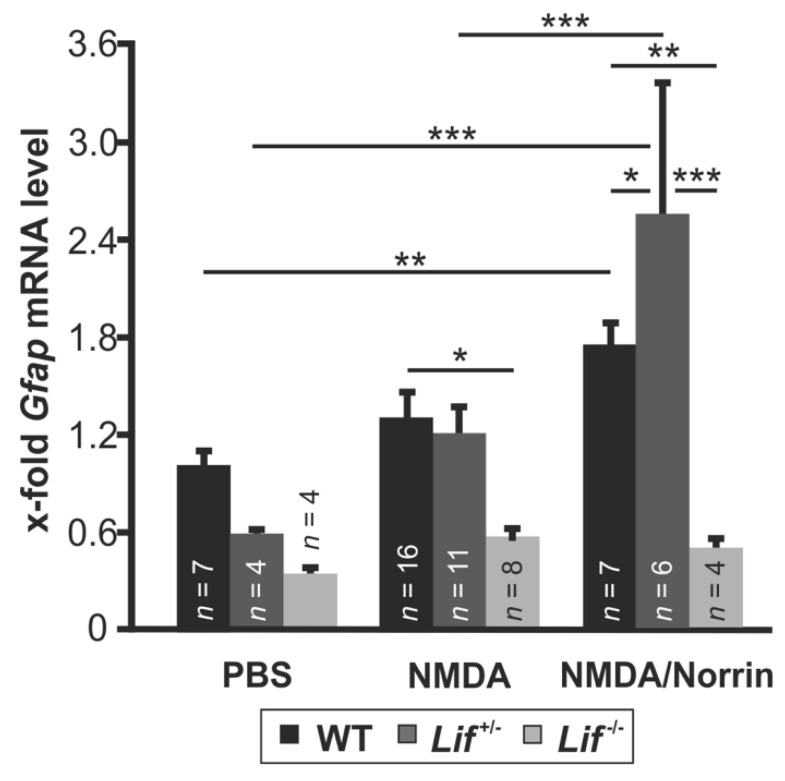
Norrin mediates Müller cell gliosis via Lif signaling. Real-time RT-PCR on retinal total RNA for Gfap from heterozygous (*Lif^+/−^*) and homozygous, Lif-deficient mice (*Lif^−/−^*), as well as wild-type (WT) littermates 7 h after intravitreal injection of 3 µL PBS, 3 µL NMDA [10mM] or 3 µL NMDA [10 mM] with Norrin [5 ng/µL]. * *p* < 0.05; ** *p* < 0.01; *** *p* < 0.001.

In wild-type mice, only a trend as well as a significant induction of Gfap mRNA was detected after treatment with NMDA (1.29 ± 0.17-fold) or NMDA plus Norrin (1.74 ± 0.16-fold), respectively, when compared to control mice (Figure 4). However, after PBS injection in hetero- and homozygous, Lif-deficient mice, a decreased Gfap mRNA expression of 0.59 ± 0.04-fold and 0.34 ± 0.05-fold, respectively, was detected when compared to PBS treated wild-type littermates (Figure 4). In contrast, the treatment of *Lif^+/−^* mice with NMDA approximately doubled the Gfap mRNA expression (1.23 ± 0.13-fold) compared to PBS controls. Furthemore, after the injection of NMDA in combination with Norrin, the mRNA levels for Gfap (2.58 ± 0.81) were significantly increased up to 4.3- and 2.1-fold, respectively, compared to PBS and NMDA treated *Lif^+/−^* mice (Figure 4). In contrast, no significant change of Gfap mRNA levels after treatment with NMDA (0.58 ± 0.08-fold) or NMDA plus Norrin (0.51 ± 0.05-fold) was detected in homozygous Lif-deficient mice (Figure 4).

### 3.5. Norrin Induces the Expression of Edn2 and Fgf2 via Lif Signaling

To investigate if Norrin mediates its protective effects via Lif signaling, the expression of the neuroprotective factors Edn2 and Fgf2 was measured following intravitreal injection of Norrin and NMDA.

**Figure 5:**
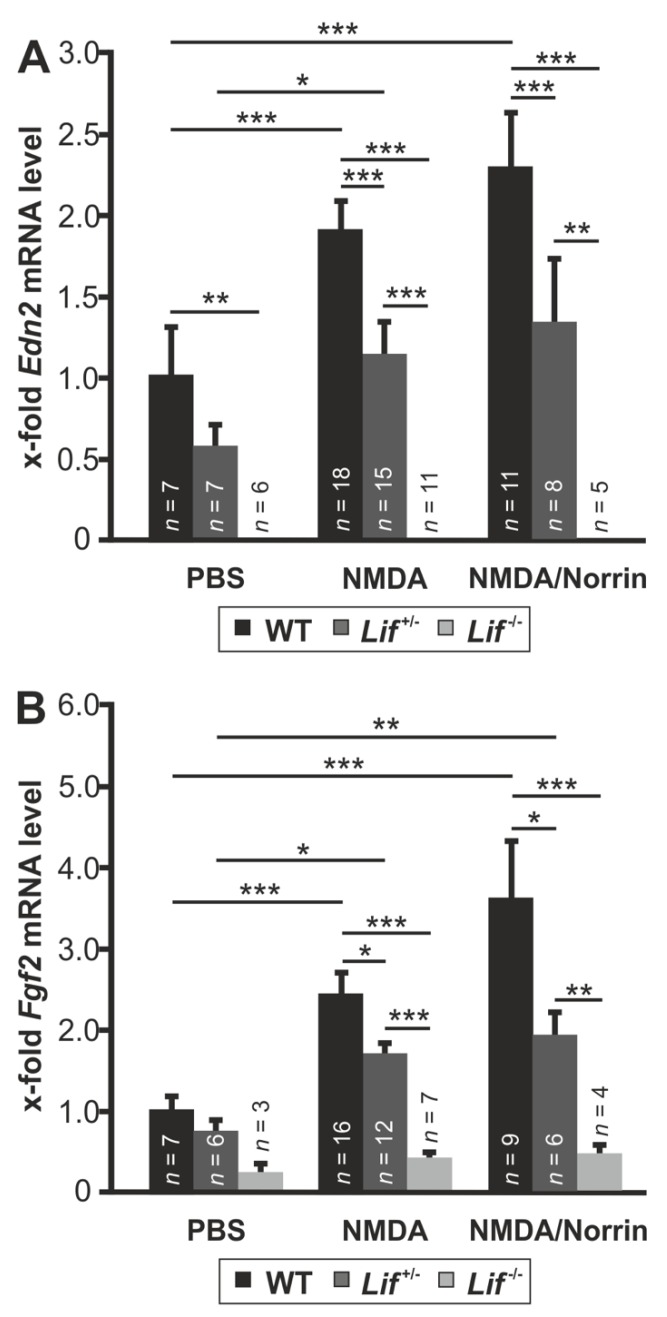
Norrin induces the expression of Edn2 and Fgf2 via Lif signaling. Real-time RT-PCR on retinal total RNA of Edn2 (**A**) and Fgf2 (**B**) from heterozygous (*Lif^+/−^*) and homozygous, Lif-deficient mice (*Lif^−/−^*), as well as wild-type (WT) littermates 7 h after intravitreal injection of 3 µL PBS, 3 µL NMDA [10 mM] or 3 µL NMDA [10 mM] with Norrin [5 ng/µL]. * *p* < 0.05; ** *p* < 0.01; *** *p* < 0.001.

In retinae of wild-type mice, the treatment with NMDA significantly enhanced the mRNA expression for Edn2 (1.88 ± 0.2-fold) and Fgf2 (2.39 ± 1.12-fold), which were further enhanced for Edn2 up to 2.27 ± 0.35-fold and for Fgf2 to 3.56 ± 0.35-fold when NMDA was injected with Norrin in comparison to PBS controls (Figure 5A). However, in heterozygous, Lif-deficient mice, the mRNA levels for Edn2 and Fgf2 following PBS injection were significantly lower 0.59 ± 0.12-fold and 0.73 ± 0.14-fold, when compared to wild-type controls (Figure 5A,B). However, treatment of heterozygous, Lif-deficient mice with NMDA led to a marked induction of Edn2 (1.14 ± 0.21-fold) and Fgf2 mRNA (1.67 ± 0.14-fold) when compared to PBS injected control littermates. When compared to NMDA-treated wild-type mice, the mRNA level for Edn2 and Fgf2 were approximately 40% and 30% lower (Figure 5A,B). In contrast, when NMDA was injected in combination with Norrin, the mRNA level for Edn2 (1.33 ± 0.41-fold) and Fgf2 (1.92 ± 0.26-fold) were slightly increased in heterozygous, Lif-deficient mice compared to NMDA-treated fellow eyes (Figure 5A,B). Again, when compared to NMDA plus Norrin injected wild-type mice, the mRNA level of Edn2 was reduced to 40% and for Fgf2 to 45% (Figure 5A,B). No signal for Edn2 mRNA and only minor levels of Fgf2 mRNA were detected in retinae of homozygous Lif-deficient mice (Figure 5A,B).

## 4. Discussion

We conclude that Lif is a central downstream molecule, which is essential in mediating the protective effects of Norrin on retinal neurons. Our conclusions are based upon three central findings: (1) the Norrin-mediated protective effect on acutely damaged retinal neurons is lost in *Lif^+/−^* and *Lif^−/−^* mice; (2) the lack of Gfap induction after combined injection of NMDA and Norrin in homozygous, Lif-deficient mice; and (3) the finding that the expression of neuroprotective factors after excitotoxic damage and Norrin injection is substantially decreased in Lif-deficient mice.

Lif has several functions in the retina, such as the formation of the retinal vascular network, to maintain its integrity [29]. Even in *Lif^−/−^* mice, the deletion of the gene is constitutive we did not to find an obvious phenotype in the retina and the optic nerve by light microscopy, an observation that is in line with previous observations [30]. Furthermore, a lack of a phenotype is assumed for 10 to 15% of all generated knockout mice, an observation that is traced back to compensating mechanisms of redundancy in gene families [31]. Ciliary neurotrophic factor (Cntf), equally to Lif, belongs to the interleukin-6 family of cytokines, which in retina activate the same downstream signaling pathways, such as Jak/STAT, ERK or AKT [22]. Analyses on degenerating motoneurons suggest that in Lif^−/−^ mice, Cntf compensates the lack of Lif and vice versa [32]. In line, in the retina of Cntf^−/−^ mice, the Lif expression was substantially increased following optic nerve crush, resulting in similar protein levels for pSTAT3, a common downstream mediator of Lif and Cntf. This effect was lost in Lif/Cntf double knockout mice [33]. Although we cannot rule out that other pathways are involved, it is most likely that an increased expression of Cntf in Lif^−/−^ mice is the leading compensatory mechanism in these animals. In keeping with literature, we confirmed the Lif deficiency in our mice by real-time RT-PCR and did not observe an obvious retinal phenotype [34,35]. Thus, we conclude that *Lif^−/−^* mice are a suitable tool to investigate the role of Lif in Norrin signaling.

By TUNEL labeling following NMDA injection, we detected apoptotic cells in the RGC layer and INL of all genotypes. This is in line with previous observations of the expression of NMDA receptors on RGCs and subtypes of amacrine cells, which are both located within these layers [36,37]. In wild-type mice, NMDA treatment substantially induced apoptotic cells in the RGC layer and INL, which was significantly reduced in both layers when Norrin was added. However, the protective effect of Norrin on retinal neurons following NMDA injection was completely lost in hetero- and homozygous Lif-deficient mice. This result strongly supports our hypothesis that Lif is a central downstream mediator of Norrin signaling. Interestingly, in Lif^+/−^ mice, after injection of NMDA, we observed more apoptotic cells in the RGC layer than in the retina of Lif^−/−^ and wild-type animals. Furthermore, in the inner nuclear layer of Lif^+/−^ as well as wild-type littermates, we found approximately twice as much TUNEL positive cells than in Lif^−/−^ mice. The increase of apoptotic cells in the RGC layer of Lif^+/−^ mice may be due to the decreased retinal expression of Lif, after acute damage of retinal neurons and moderate knockout-induced compensatory mechanisms in this genotype. Since amacrine cells are more sensitive to excitotoxic damage than RGC [38,39] it is tempting to speculate whether the potential protective effects of Lif on amacrine cells in wild-type mice are covered by an excessive NMDA-induced damage on these cells. In turn, this could explain the similar number of TUNEL positive cells in wild-type and Lif^+/−^ mice. However, in Lif^−/−^ mice, after NMDA injection, the number of apoptotic cells in the RGC layer was similar to that of wild-type mice and approximately halved in the INL when compared to Lif^+/−^ and wild-type controls. As discussed above, it is likely that in Lif^−/−^ mice, the lack of Lif is compensated by an enhanced expression of Cntf. For Cntf, a substantial protection of RGC and amacrine cells was shown following optic nerve crush or induction of NMDA-mediated excitotoxic retinal damage, respectively [40,41]. Therefore, it may be possible that a knockout-induced, increased compensatory expression of Cntf in Lif^−/−^ mice can mediate a more pronounced protective effect on retinal neurons following excitotoxic damage than the delayed induction of Lif in wild-type mice. Overall, after injection of NMDA with Norrin into eyes of Lif^+/−^ and Lif^−/−^ mice, the protective effect of Norrin on retinal neurons was blocked, strongly suggesting that Norrin mediates its protective effects, following acute excitotoxic damage via the induction of Lif.

Following retinal damage, morphological, biochemical and physiological alterations in Müller cells take place, a response called gliosis reaction, which can result in protective but damaging effects for retinal neurons [42]. Gfap is known as a marker of Müller cell activation, which has an enhanced expression in retinal degeneration or following acute excitotoxic damage in RGC and amacrine cells [17,28,42]. Subsequent after injection of NMDA or NMDA plus Norrin, we found a slight or a significant induction of Gfap mRNA in wild-type as well as in heterozygous, Lif-deficient mice. However, following injection of NMDA with Norrin, the mRNA expression for Gfap was approximately 40% higher in Lif^+/−^ mice than in wild-type animals. This may be due to additional factors inducing Gfap expression or the variability of gene expression in heterozygous knockout mice. In contrast, in homozygous, Lif-deficient mice the NMDA as well as the NMDA plus Norrin-mediated effect on Gfap expression was lost. This observation is in line with previous reports of Lif-mediated gliosis reactions following acute damage of photoreceptors [19]. Overall, our data strongly suggests that an enhanced Lif expression is required to promote the Norrin-mediated gliosis reaction in Müller cells following excitotoxic damage. For degenerating photoreceptors, a self-enhancing signaling network involving the expression of Lif in Müller cells and End2 in photoreceptors has been proposed, which in turn leads to an enhanced gliosis reaction and to an induction of neuroprotective factor [19]. In line with this signaling network, we detected an enhanced expression of Lif, End2 and Fgf2 in wild-type mice following NMDA or NMDA plus Norrin injection, which have the distinct potential to mediate protective effects on damaged retinal neurons [43,44]. This induction was significantly decreased or blocked in Lif-deficient mice. The lacking End2 mRNA expression and low Fgf2 mRNA levels in Lif-deficient mice strongly suggests a central role of Lif for downstream signaling of the neuroprotective effects of Norrin.

In our current study, we found an enhanced expression of Lif after treatment of wild-type mice retinae with NMDA and Norrin. However, in a mouse model with a chronic degeneration of RGC and an additional overexpression of Norrin in the retina, we also observed a protective effect of Norrin but no induction of Lif [45]. It is most likely, that other factors could be required for the Norrin-mediated induction of Lif after acute excitotoxic retinal damage. Thus, the identification of these mediators would further clarify this mechanism in retinae. In line, depending on cellular and environmental conditions, Lif mediates its functions predominantly via an activation of the STAT, ERK or AKT signaling pathway [46]. Discovering potential downstream mediators of the Norrin-mediated protection of retinal neurons via Lif, would further contribute to the understanding of this signaling pathway. Finally, after induction of retinal excitotoxic damage, the role of reactive microglia is being discussed [47,48]. Therefore, the exploration of the effect of Norrin on microglia cells following acute retinal damage would open novel insights in Norrin-mediated neuroprotection of the retina.

Overall, our data strongly indicate that following excitotoxic retinal damage, Norrin mediates its protective effects on retinal neurons via an induction of Lif, which appears to be a central signaling molecule to promote Müller cell gliosis and induction of protective factors such as End2 or Fgf2.

## Figures and Tables

**Table 1:** Primers used for real-time reverse transcription-polymerase chain reaction (RT-PCR) amplification.

Gene	Sequence (Forward)	Sequence (Reverse)
Mouse primers		
Edn2	5′-ACCTCCTCCGAAAGCTGAG-3′	5′-TTTCTTGTCACCTCTGGCTGTA-3°
Fgf2	5′-CGGCTCTACTGCAAGAACG-3′	5′-TGCTTGGAGTTGTAGTTTGACG-3′
Gfap	5′-ACAGACTTTCTCCAACCTCCAG-3′	5′-CCTTCTGACACGGATTTGGT-3′
Gnb2l	5′-TCTGCAAGTACACGGTCCAG-3′	5′-GAGACGATGATAGGGTTGCTG-3′
Lif	5′-AAACGGCCTGCATCTAAGG-3′	5′-AGCAGCAGTAAGGGCACAAT-3′
